# The expression patterns and the diagnostic/prognostic roles of PTPN family members in digestive tract cancers

**DOI:** 10.1186/s12935-020-01315-7

**Published:** 2020-06-12

**Authors:** Jing Chen, Xu Zhao, Yuan Yuan, Jing-Jing Jing

**Affiliations:** 1 Tumor Etiology and Screening Department of Cancer Institute and General Surgery, The First Hospital of China Medical University, No. 155 North NanjingBei Street, Heping District, Shenyang, 110001 Liaoning People’s Republic of China; 2 Key Laboratory of Cancer Etiology and Prevention in Liaoning Education Department, The First Hospital of China Medical University, Shenyang, 110001 China; 3 Key Laboratory of GI Cancer Etiology and Prevention in Liaoning Province, The First Hospital of China Medical University, Shenyang, 110001 China; 4 Mathematical Computer Teaching and Research Office, Liaoning Vocational College of Medicine, Shenyang, 110101 China

**Keywords:** PTPN family members, Digestive tract cancers, Expression, Diagnosis, Prognosis, Clinical features

## Abstract

**Background:**

Non-receptor protein tyrosine phosphatases (PTPNs) are a set of enzymes involved in the tyrosyl phosphorylation. The present study intended to clarify the associations between the expression patterns of PTPN family members, and diagnosis as well as the prognosis of digestive tract cancers.

**Methods:**

Oncomine and Ualcan were used to analyze PTPN expressions. Data from The Cancer Genome Atlas (TCGA) were downloaded through UCSC Xena for validation and to explore the relationship of the PTPN expression with diagnosis, clinicopathological parameters and survival of digestive tract cancers. Gene ontology enrichment analysis was conducted using the DAVID database. The gene–gene interaction network was performed by GeneMANIA and the protein–protein interaction (PPI) network was built using STRING portal coupled with Cytoscape. The expression of differentially expressed PTPNs in cancer cell lines were explored using CCLE. Moreover, by histological verification, the expression of four PTPNs in digestive tract cancers were further analyzed.

**Results:**

Most PTPN family members were associated with digestive tract cancers according to Oncomine, Ualcan and TCGA data. Several PTPN members were differentially expressed in digestive tract cancers. For esophageal carcinoma (ESCA), PTPN1 and PTPN12 levels were correlated with incidence; PTPN20 was associated with poor prognosis. For stomach adenocarcinoma (STAD), PTPN2 and PTPN12 levels were correlated with incidence; PTPN3, PTPN5, PTPN7, PTPN11, PTPN13, PTPN14, PTPN18 and PTPN23 were correlated with pathological grade; PTPN20 expression was related with both TNM stage and N stage; PTPN22 was associated with T stage and pathological grade; decreased expression of PTPN5 and PTPN13 implied worse overall survival of STAD, while elevated PTPN6 expression indicated better prognosis. For colorectal cancer (CRC), PTPN2, PTPN21 and PTPN22 levels were correlated with incidence; expression of PTPN5, PTPN12, and PTPN14 was correlated with TNM stage and N stage; high PTPN5 or PTPN7 expression was associated with increased hazards of death. CCLE analyses showed that in esophagus cancer cell lines, PTPN1, PTPN4 and PTPN12 were highly expressed; in gastric cancer cell lines, PTPN2 and PTPN12 were highly expressed; in colorectal cancer cell lines, PTPN12 was highly expressed while PTPN22 was downregulated. Results of histological verification experiment showed differential expressions of PTPN22 in CRC, and PTPN12 in GC and CRC.

**Conclusions:**

Members of PTPN family were differentially expressed in digestive tract cancers. Correlations were found between PTPN genes and clinicopathological parameters of patients. Expression of PTPN12 was upregulated in both STAD and CRC, and thus could be used as a diagnostic biomarker. Differential expression of PTPN12 in GC and CRC, and PTPN22 in CRC were presented in our histological verification experiment.

## Background

Tyrosyl phosphorylation is a dynamic and reversible process which plays a pivotal part in many cellular signaling pathways [1]. The dephosphorylation of tyrosine residues are catalyzed by a series of enzymes named protein tyrosine phosphatases (PTPs) [2]. Encoded by 103 genes, PTPs are sorted into four major superfamily classes [3], and every single PTP was denominated an official gene name by The Human Genome Organization in Nomenclature Committee. According to the latter system, 17 non-receptor PTPs which belong to the biggest family class I, are designated PTPN, followed by a number [3]. There is mounting evidence suggesting that the cross-talk of the PTPN gene family members is involved in extensive physiological processes, such as cell proliferation, survival, immune response, migration, and metabolism [3–6]. Previously published study stated that PTPN family members play an essential part in numerous diseases. For example, the expression of PTPN6 with the loss of pSTAT3 expression could be chosen as a biomarker for the prognosis of peripheral-T cell lymphoma [7]. One research published recently noted that a deficiency of PTPN2 could enhance anti-tumor immunity and the therapeutic efficacy of CAR T cells to solid cancers [8]. PTPN22 plays an important part in regulating autophagy and NLR family pyrin domain containing 3 inflammasome activation [9]. It is hopeful that PTPN genes have potential to be served as prognostic and diagnostic indicators [10], and even therapeutic targets.

As main malignancies of gastrointestinal tract, esophagus cancer (EC), gastric cancer (GC) and colorectal cancer (CRC) are responsible for a large portion of cancer-related deaths worldwide, of which the data are 5.3%, 8.2% and 9.0% respectively [11]. All of the three digestive tract cancers are ranked in the top 10 for incidence rates of tumors and have poor prognosis [11]. Although a couple of diagnostic biomarkers have been observed, robust biomarkers to predict clinical outcomes are still in urgent need [12]. Former investigations reported that the expression patterns of individual PTPNs and its correlations with patients in various digestive tract neoplasms, but research to date has not yet observed the whole picture of the entire PTPN family, from the aspects of diagnosis, prognosis and expression characteristics. Personalized therapies based upon the genetics of individual cancer will be prior to other treatments in the near future. This research therefore is intended to illuminate the clinical value of different PTPN genes to support potential biomarkers and new individualized targets for patients. In the current study, we analyzed the expression status of different PTPN members and their diagnostic and prognostic values to comprehensively evaluate the role of PTPNs in various digestive tract cancers.

## Methods

### Oncomine database analysis for the expression patterns of PTPN family in digestive tract cancers

Oncomine database is a cancer microarray database (https://www.oncomine.org/) which shows the expression information of genes in cancer and normal samples [13]. Oncomine provides both microarray information from 715 datasets and a set of online data-mining functions. The expression levels of individual PTPN family members in different types of cancer were obtained from the Oncomine database. Student’s *t* test was applied to calculate the *P* value for expression differences of PTPN family genes between normal controls and cancer samples. The threshold parameters of *P* value and fold change were demarcated as 0.05 and 2 respectively.

### Ualcan database analysis for the validation

Ualcan is a publicly available web-portal (http://ualcan.path.uab.edu) that offers online analysis of data from The Cancer Genome Atlas (TCGA) [14]. In this study, we used it to analyze the relative expression of PTPN family genes in esophageal carcinoma (ESCA), stomach adenocarcinoma (STAD), colon adenocarcinoma (COAD), rectum adenocarcinoma (READ) and normal samples. The expression level of PTPN members was normalized as transcript per million reads, and a *P* value of no more than 0.01 conducted through Student’s *t* test was considered to be significant.

### TCGA data obtainment through UCSC Xena

The Cancer Genome Atlas (TCGA) is a cancer genomics database (https://cancergenome.nih.gov/) which contains genomic information of over 2000 primary neoplasms and matched normal samples [15, 16]. In our study, case information concerning the mRNA expression profiles and clinical features were obtained from TCGA database, which was downloaded through the University of California Santa Cruz Xena (UCSC Xena; https://xena.ucsc.edu/) platform [17]. Data of 184 ESCA and 11 matched normal samples, 415 STAD and 35 matched normal samples, and 380 CRC and 51 matched normal samples were extracted for further analysis.

### Analysis of PTPN members in diagnosis and prognosis of digestive tract cancers

Receiver operating characteristic (ROC) models were established to evaluate the diagnostic values of the differentially expressed PTPNs according to Oncomine and Ualcan analysis. SPSS software version 23.0 (IBM, SPSS, and Chicago, IL, USA) was applied to plot ROC curves. *P *< 0.01 was considered significant. The Pearson X^2^ test was performed to evaluate the relationship between PTPN expression status and clinicopathological parameters including TNM stage and grade. All of the patients were differentiated into low expression and high expression groups using the third quartile of mRNA expressions as the cut-off value. The effects of different PTPN expressions on overall survival (OS) were estimated through univariate and multivariate Cox proportional hazards models with or without adjustment for confounding factors. Variables including gender, TNM stage, and age were further adjusted during the evaluation. The medical data obtained was managed by R language (Version 3.6.1). *P* value < 0.05 was considered statistically significant.

### GO and PPI analysis for function and interaction of PTPN family

Enrichment analysis of Gene Ontology (GO) of PTPN genes was explored using the Database for Annotation, Visualization and Integrated Discovery (DAVID; v.6.8; https://david.ncifcrf.gov/home.jsp; accessed on November 20, 2019) [18]. The gene–gene interaction network was structured using the Gene Multiple Association Network Integration Algorithm (GeneMANIA; https://www.genemania.org/; accessed on November 21, 2019) [19] and the Search Tool for the Retrieval of Interacting Genes Database (STRING v.10.0; https://string-db.org/; accessed on November 23, 2019) was used to create a protein–protein interaction (PPI) network [20]. The Cytoscape software was applied to visualize network diagrams for PPI analysis [21].

### Evaluation of PTPN expression in cancer cell lines by CCLE analysis

The Broad Institute Cancer Cell Line Encyclopedia (CCLE; http://www.broadinstitute.org/ccle) is a database, which aims to provide available access to detailed genomic information, computational analyses and visualization for over 1100 cell lines representing 37 cancer types [22]. The expression levels of PTPN1, PTPN2, PTPN4, PTPN12, PTPN21 and PTPN22 in different types of cancer cell lines were investigated through CCLE database.

### Collection of specimens and histological verification experiment

All clinical specimens used to measure mRNA levels of PTPN1, PTPN2, PTPN12 and PTPN22 were collected from the First Hospital of China Medical University, Shenyang, China. The study was approved by the ethics committee of the First Hospital of China Medical University. The written informed consent has been obtained from each participant before specimen collection. Specimens were collected in accordance with the Declaration of Helsinki and legal regulations.

Total RNA was extracted from 30 pairs of patient tissues (10 EC, 10 GC and 10 CRC) and adjacent non-tumor tissues. Relative mRNA expression levels were detected by quantitative real-time PCR (qRT-PCR).The qRT-PCR experiment was performed with a real-time PCR 480 system and SYBR-green PCR Master Mix. All of the qRT-PCR curves were with single peak. *P* < 0.05 was considered to imply significant results. Primer sequences for PTPN members and beta-actin were tabulated in Additional file 1: Table S1.

## Results

### PTPN genes expression patterns in digestive tract cancers

**Fig. 1:**
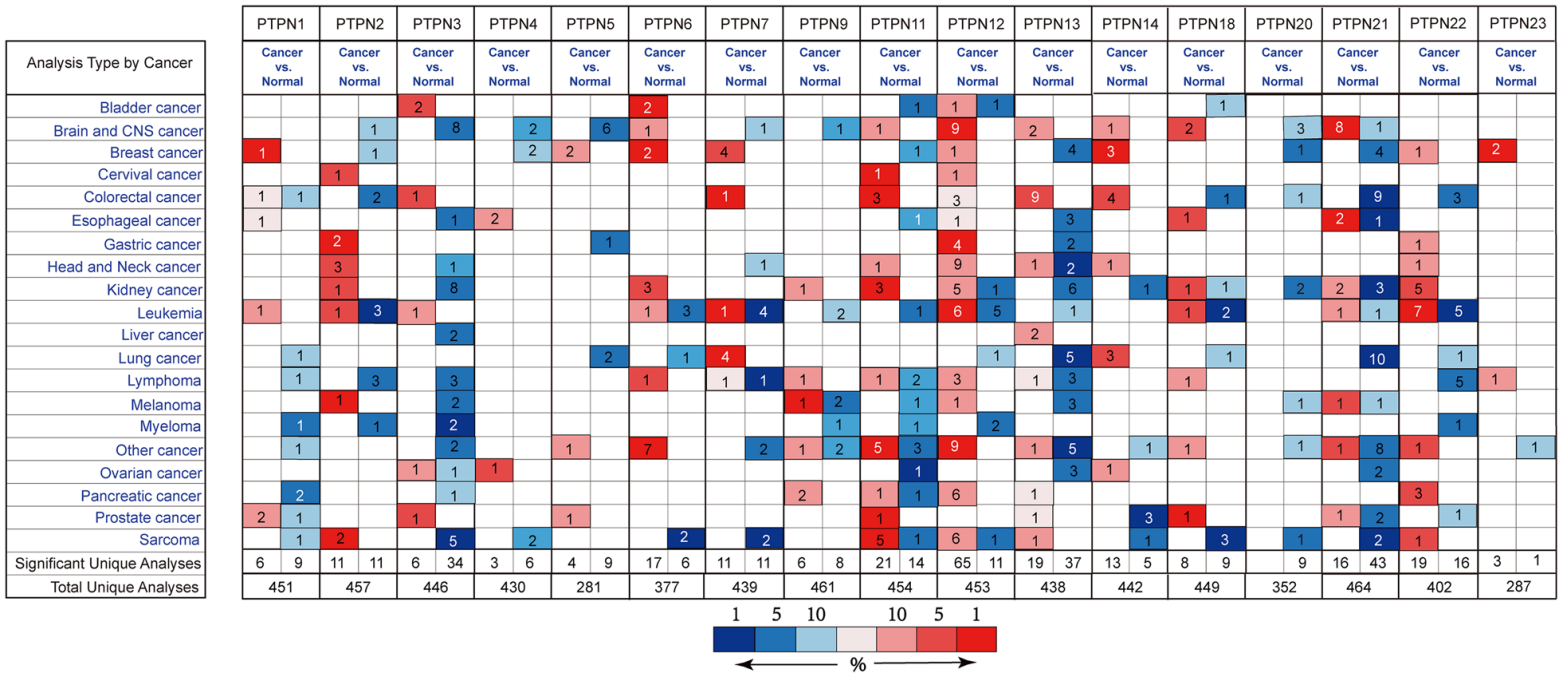
The expression level of PTPN family genes in different types of cancers. Red and blue stand for the numbers of datasets with statistically significant (P < 0.05) increased and decreased levels of PTPN family genes, respectively

Before expression profiling analysis, we refined the chromosome location of all PTPN members through published literature review. The detailed information was summarized in Table 1. All of the PTPN family members have been analyzed in our study. According to the analysis results of Oncomine, the expression of PTPN genes were different in all types of cancer and its matched normal tissues (Fig. 1). For EC samples, PTPN1, PTPN4, PTPN12, PTPN18 and PTPN21 were over-expressed, while PTPN3, PTPN11, PTPN13 and PTPN21 were downregulated. In GC tissues, the expression of PTPN5 and PTPN13 was decreased while at the same time PTPN2, PTPN12 and PTPN22 were highly expressed in patients. As for CRC patients, the expression of PTPN1, PTPN3, PTPN7, PTPN11, PTPN12, PTPN13 and PTPN14 was upregulated, while PTPN2, PTPN18, PTPN20, PTPN21 and PTPN22 were expressed in a lower level in tissues. Comprehensively, the expression of PTPN12 was higher in EC, GC and CRC samples.

**Table 1:** Basic characteristics of PTPN family genes

HGNC ID (gene)	Gene ID	Approved symbol	Synonym(s)	Exon	Chromosomal location
HGNC:9642	5770	PTPN1	PTP1B	10	20q13.13
HGNC:9650	5771	PTPN2	TCELLPTP	15	18p11.21
			TC-PTP		
			TCPTP		
HGNC:9655	5774	PTPN3	PTPH1	33	9q31
HGNC:9656	5775	PTPN4	PTPMEG	29	2q14.2
HGNC:9657	84867	PTPN5	STEP	16	11p15.1
			PTPSTEP		
			STEP61		
HGNC:9658	5777	PTPN6	HCP	17	12p13.31
			HCPH		
			PTP-1C		
			SHP-1		
			SHP1		
HGNC:9659	5778	PTPN7	HEPTP	12	1q32.1
			LC-PTP		
HGNC:9661	5780	PTPN9	MEG2	13	15q24.2
HGNC:9644	5781	PTPN11	BPTP3	16	12q24.13
			SH-PTP2		
			SHP-2		
			PTP2C		
			SHP2		
HGNC:9645	5782	PTPN12	PTPG1	19	7q11.23
			PTP-PEST		
HGNC:9646	5783	PTPN13	PTP1E	48	4q21.3
			PTP-BAS		
			PTPL1		
			PTP-BL		
HGNC:9647	5784	PTPN14	PEZ	21	1q32.3-q41
HGNC:9649	26469	PTPN18	BDP1	15	2q21.1
HGNC:23423	26095	PTPN20	bA42B19.1	18	10q11.22
			DKFZP566K0524		
			bA142I17.1		
			CT126		
HGNC:9651	11099	PTPN21	PTPD1	23	14q31
			PTPRL10		
HGNC:9652	26191	PTPN22	Lyp	24	1p13.2
			Lyp1		
			Lyp2		
HGNC:14406	25930	PTPN23	DKFZP564F0923	25	3p21.31
			KIAA1471		
			HD-PTP

**Fig. 2:**
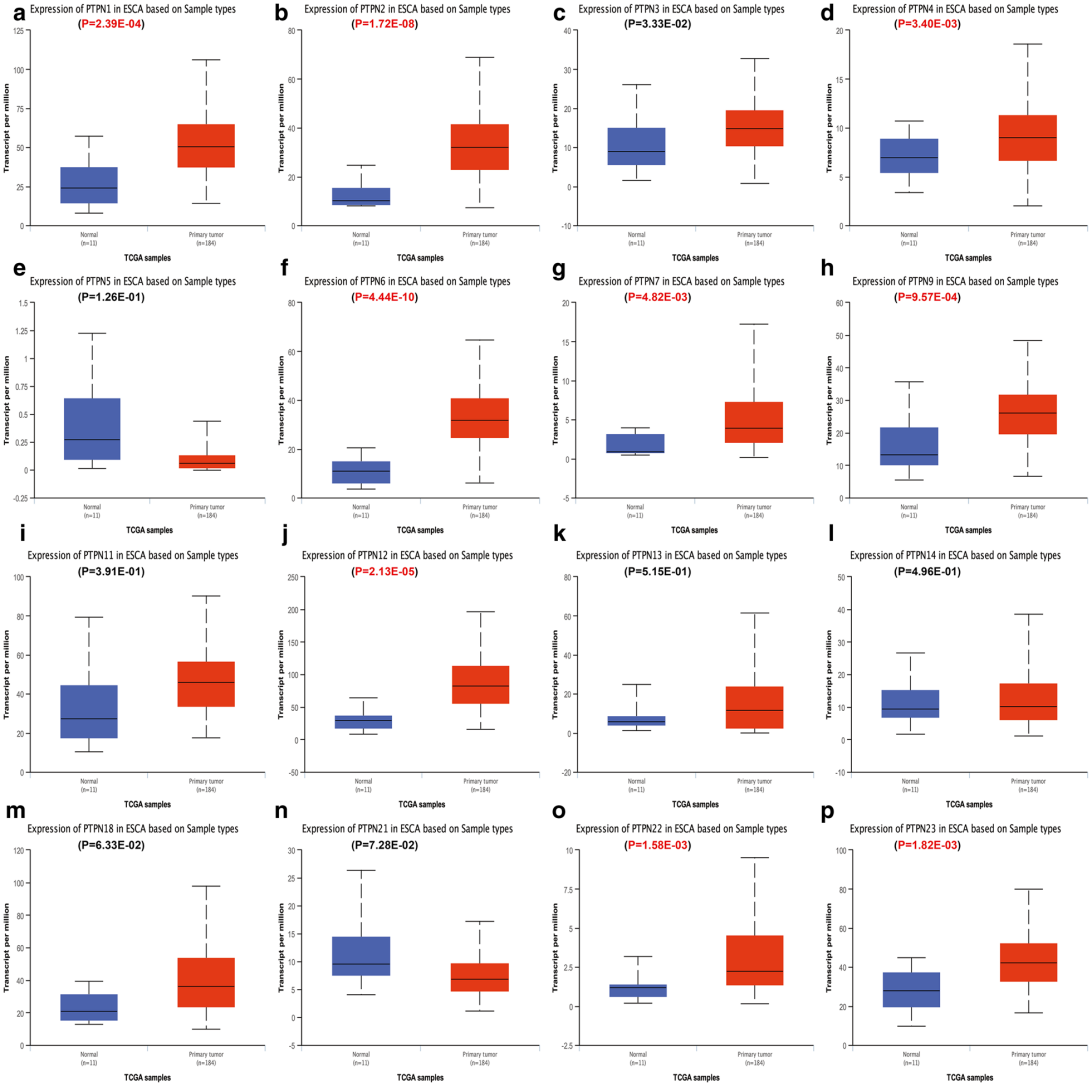
The relative expression of PTPN genes in normal tissues and esophageal carcinoma tissues. **a** PTPN1, **b** PTPN2, **c** PTPN3, **d** PTPN4, **e** PTPN5, **f** PTPN6, **g** PTPN7, **h** PTPN9, **i** PTPN11, **j** PTPN12, **k** PTPN13, **l** PTPN14, **m** PTPN18, **n** PTPN21, **o** PTPN22, **p** PTPN23

**Fig. 3:**
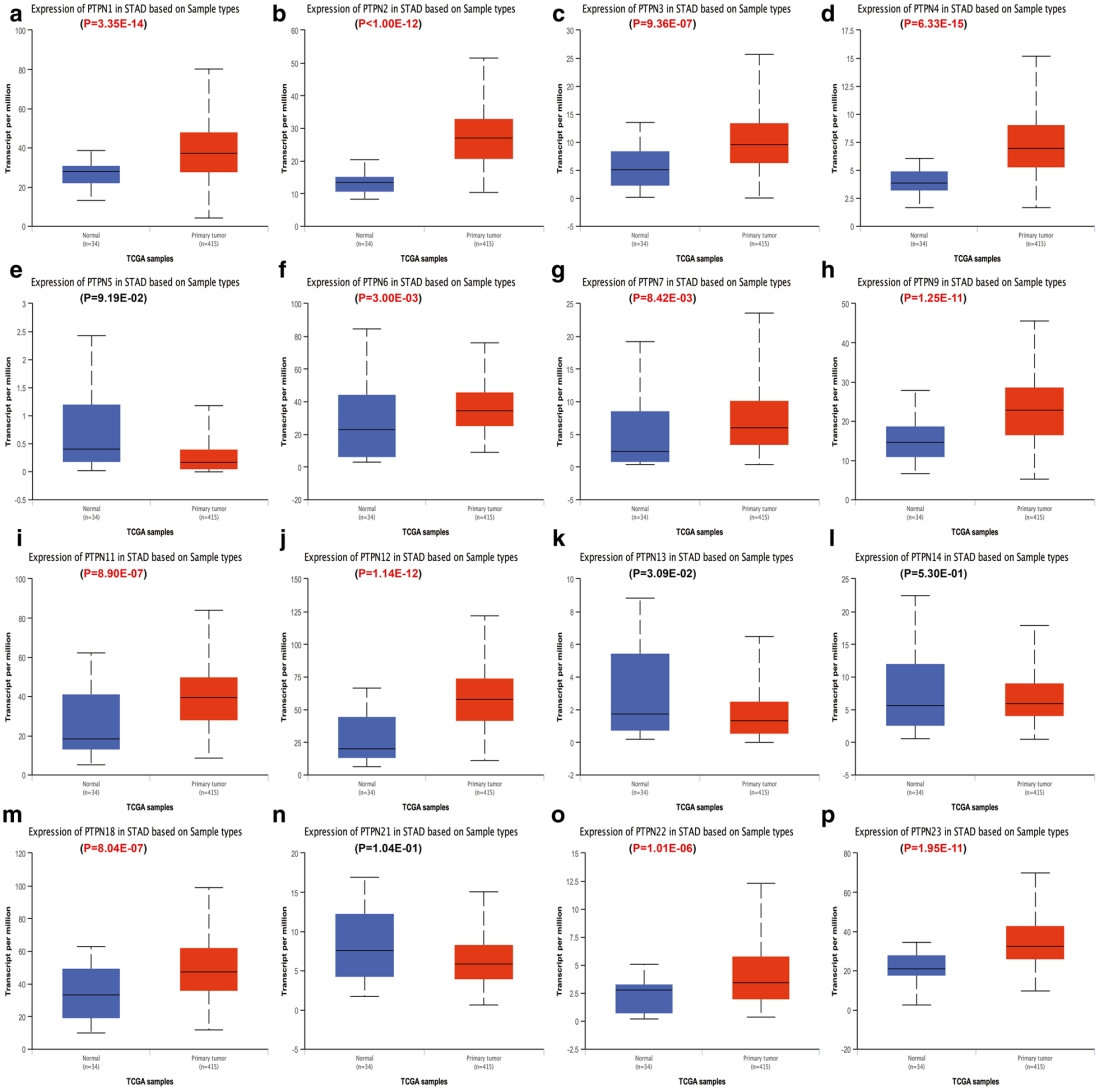
The relative expression of PTPN genes in normal tissues and stomach adenocarcinoma tissues. **a** PTPN1, **b** PTPN2, **c** PTPN3, **d** PTPN4, **e** PTPN5, **f** PTPN6, **g** PTPN7, **h** PTPN9, **i** PTPN11, **j** PTPN12, **k** PTPN13, **l** PTPN14, **m** PTPN18, **n** PTPN21, **o** PTPN22, **p** PTPN23

**Fig. 4:**
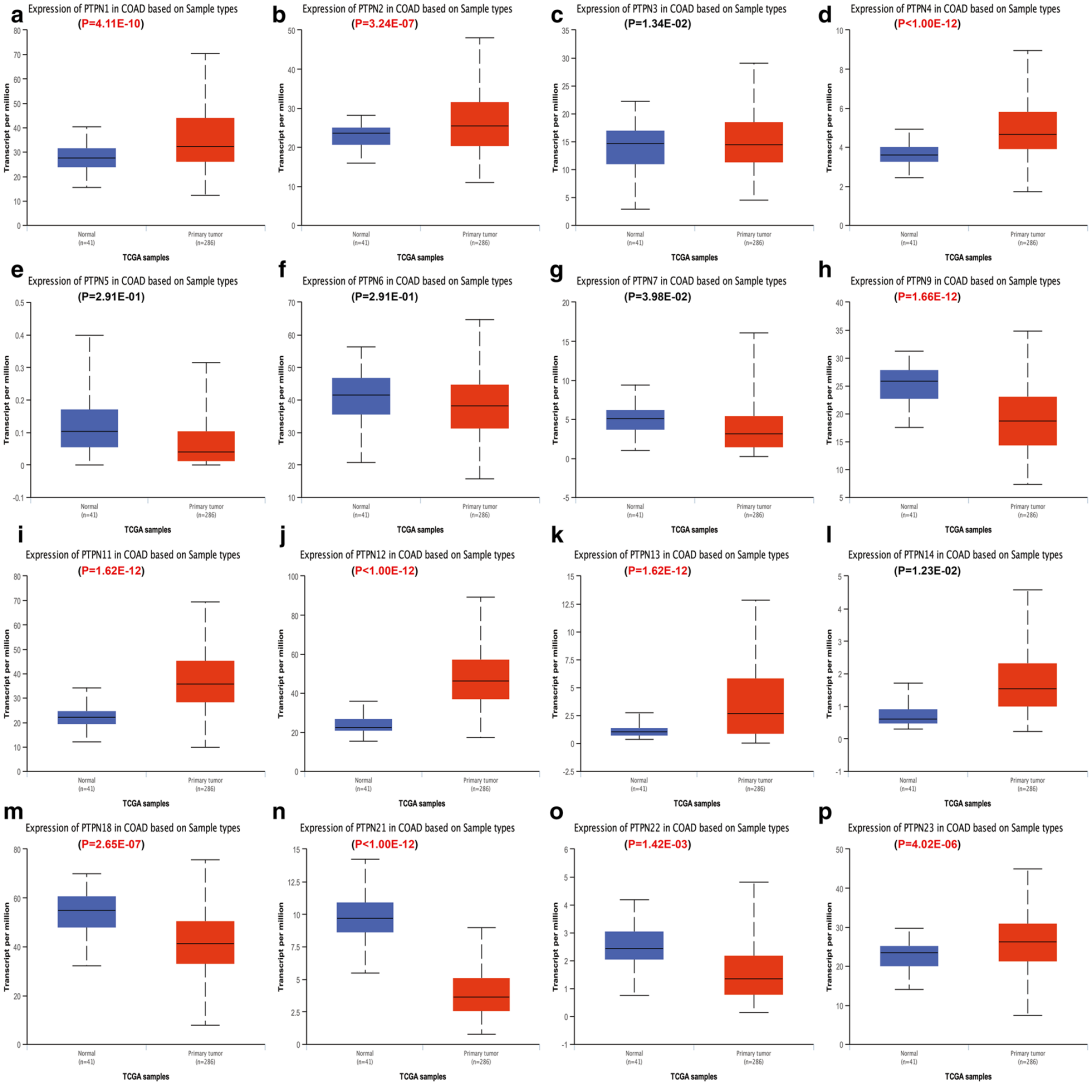
The relative expression of PTPN genes in normal tissues and colon adenocarcinoma tissues. **a** PTPN1, **b** PTPN2, **c** PTPN3, **d** PTPN4, **e** PTPN5, **f** PTPN6, **g** PTPN7, **h** PTPN9, **i** PTPN11, **j** PTPN12, **k** PTPN13, **l** PTPN14, **m** PTPN18, **n** PTPN21, **o** PTPN22, **p** PTPN23

**Fig. 5:**
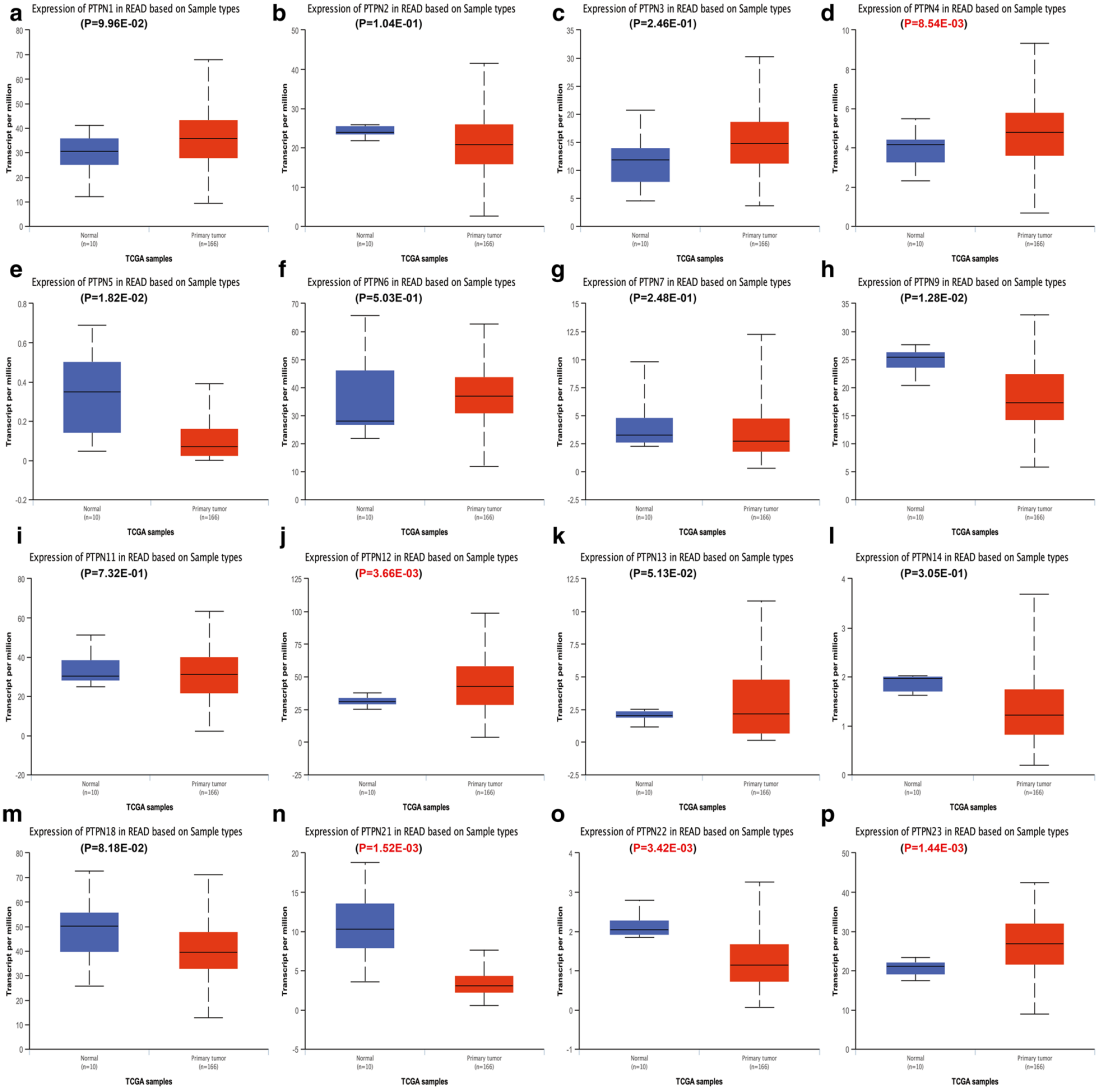
The relative expression of PTPN genes in normal tissues and rectum adenocarcinoma tissues. **a** PTPN1, **b** PTPN2, **c** PTPN3, **d** PTPN4, **e** PTPN5, **f** PTPN6, **g** PTPN7, **h** PTPN9, **i** PTPN11, **j** PTPN12, **k** PTPN13, **l** PTPN14, **m** PTPN18, **n** PTPN21, **o** PTPN22, **p** PTPN23

Then we used Ualcan database to further verify the expression of PTPNs among ESCA, STAD, COAD, READ and their matched normal tissues in the TCGA datasets. As shown in the results of Ualcan (Figs. 2, 3, 4, 5), there was a clear trend that the expression levels of PTPN1, PTPN2, PTPN4, PTPN6, PTPN7, PTPN9, PTPN12, PTPN22 and PTPN23 were statistically higher in samples of ESCA than in normal tissues; in STAD, PTPN1, PTPN2, PTPN3, PTPN4, PTPN6, PTPN7, PTPN9, PTPN11, PTPN12, PTPN18, PTPN22, and PTPN23 were significantly increased compared to normal samples; in COAD, PTPN1, PTPN2, PTPN4, PTPN11, PTPN12, PTPN13, and PTPN23 were overexpressed compared with normal samples, while PTPN9, PTPN18, PTPN21 and PTPN22 were lower than normal tissues; in READ, the expression levels of PTPN4, PTPN12, and PTPN23 were higher than normal samples while PTPN21 and PTPN22 were decreased in patients compared to normal groups. Taken together, the results above indicated that expression status of PTPN4, PTPN12 and PTPN23 was upregulated in ESCA, STAD, COAD and READ. PTPN21 and PTPN22 were upregulated in ESCA and STAD but downregulated in COAD and READ.

### The diagnostic efficiency of PTPN genes in digestive tract cancers

According to the expression analysis of Oncomine and Ualcan above, some PTPN genes were over-expressed in digestive tract cancers, while others were downregulated. Therefore, we computed ROC curves to further analyze the diagnostic efficiency of these differentially expressed PTPNs. The results revealed that upregulation of PTPN1 (Area = 0.784, 95% CI 0.675–0.893, and P < 0.002) and PTPN12 (Area = 0.860, 95% CI 0.768–0.951, and P < 0.001) was correlated with ESCA incidence; upregulation of PTPN2 (Area = 0.839, 95% CI 0.771–0.906, and P < 0.001) and PTPN12 (Area = 0.775, 95% CI 0.680–0.871, and P < 0.001) was associated with STAD incidence; and upregulation of PTPN12 (Area = 0.946, 95% CI 0.921–0.970, and P < 0.001), and downregulation of PTPN21 (Area = 0.986, 95% CI 0.977–0.995, and P < 0.001) and PTPN22 (Area = 0.764, 95% CI 0.704–0.825, and P < 0.001) were associated with CRC incidence (Table 2). Detailed results of the ROC curves are shown in Additional file 2. Our results above suggest that levels of these genes could be exploited as effective biomarkers for the diagnosis of specific digestive tract cancers. Especially, PTPN12 might serve as a useful diagnostic biomarker for both ESCA, STAD and CRC.

**Table 2:** The ROC test results of PTPN genes in digestive tract cancers

Cancer type	Gene	Area	P value	95% confidence interval	
				Lower bound	Upper bound
ESCA	PTPN1	0.784091	*0.00156*	0.67476	0.893422
ESCA	PTPN4	0.384387	0.1981	0.179313	0.589461
ESCA	PTPN12	0.859684	*0.00006*	0.76844	0.950928
STAD	PTPN2	0.838554	**<***0.00001*	0.771215	0.905894
STAD	PTPN12	0.775387	**<***0.00001*	0.680006	0.870769
STAD	PTPN22	0.59642	0.05802	0.49601	0.69683
CRC	PTPN12	0.945511	**<***0.00001*	0.921075	0.969947
CRC	PTPN21	0.98612	**<***0.00001*	0.976818	0.995421
CRC	PTPN22	0.764241	**<***0.00001*	0.703726	0.824757

Significant P values are expressed in Italics

*ROC* receiver operating characteristics

### The correlations between PTPN genes and clinicopathological parameters

Association of PTPN genes expression status with different clinicopathological features of ESCA, STAD and CRC were analyzed in the study. For ESCA, no statistically association was found between PTPN members and clinicopathological parameters (Additional file 1: Table S2). For STAD, grade was significantly correlated with PTPN3, PTPN11, PTPN13, PTPN14, and PTPN18 (P = 0.015, 0.021, 0.005, 0.002 and 0.034, resp.; Additional file 1: Table S3). In addition, PTPN5, PTPN7 and PTPN23 showed statistically association with pathological grade of STAD with a p value of no more than 0.001. Besides, PTPN20 expression was related with TNM stage (P = 0.002) and N stage (P = 0.038); PTPN22 was significantly related with grade (P < 0.001) and T stage (P = 0.039) (Additional file 1: Table S3). For CRC, TNM stage was correlated with expression of PTPN5, PTPN12, and PTPN14 (P = 0.049, 0.027, 0.002, resp.; Additional file 1: Table S4); N stage was associated with expression of PTPN5, PTPN12, and PTPN14 (P = 0.034, 0.018 and 0.004, resp.; Additional file 1: Table S4). According to the results above, PTPN5 and PTPN14 were associated with the clinicopathological parameters of both STAD and CRC.

### The prognostic roles of PTPN genes in digestive tract cancers

The analysis results of the relationship between PTPN family genes and the prognosis of digestive tract cancers showed that for ESCA, elevated expression of PTPN20 was associated with a worse overall survival (OS) of ESCA in multivariate model (P = 0.038, adjusted HR = 1.982, 95% CI 1.039–3.780; Table 3). For STAD, decreased PTPN5 expression was associated with worse OS according to univariate analysis (P = 0.006, HR = 1.680, 95% CI 1.162–2.429) and multivariate analysis (P = 0.004, adjusted HR = 1.753, 95% CI 1.192–2.577), while elevated expression of PTPN6 indicated longer OS based on the results of univariate analysis (P = 0.028, HR = 0.633, 95% CI 0.421–0.951) and multivariate analysis (P = 0.017, adjusted HR = 0.606, 95% CI 0.402–0.915) (Table 4). Besides, univariate analysis exhibited that there was a significant association between decreased PTPN13 expression and decreased OS (P = 0.036, HR = 1.471, 95% CI 1.026–2.109). CRC patients with high PTPN5 expression showed increased hazards of death in univariate model (P = 0.021, HR = 1.947, 95% CI 1.105–3.431; Table 5). Elevated expression of PTPN7 was significantly associated with the unfavorable OS for every CRC patients according to multivariate analysis (P = 0.013, adjusted HR = 2.043, 95% CI 1.164–3.584; Table 5). From the results above we can see that the expression of PTPN5 was associated with the prognosis of both STAD and CRC.

**Table 3:** Prognostic role of PTPN family genes in ESCA

Gene	Univariate analysis		Multivariate analysis	
	HR (95% CI)	P	Adjusted HR (95% CI)	P
PTPN1	0.831 (0.427–1.615)	0.585	0.724 (0.367–1.427)	0.35
PTPN2	1.171 (0.601–2.285)	0.643	1.075 (0.546–2.119)	0.834
PTPN3	1.020 (0.514–2.026)	0.954	0.776 (0.383–1.571)	0.481
PTPN4	0.617 (0.304–1.253)	0.182	0.758 (0.348–1.648)	0.484
PTPN5	0.982 (0.494–1.952)	0.958	0.935 (0.452–1.933)	0.855
PTPN6	1.123 (0.565–2.230)	0.741	1.398 (0.678–2.886)	0.364
PTPN7	0.839 (0.432–1.630)	0.605	0.817 (0.394–1.693)	0.586
PTPN9	1.499 (0.774–2.904)	0.23	1.302 (0.666–2.546)	0.44
PTPN11	0.632 (0.293–1.362)	0.241	0.597 (0.275–1.294)	0.191
PTPN12	0.706 (0.356–1.399)	0.319	0.518 (0.254–1.057)	0.071
PTPN13	0.695 (0.332–1.453)	0.334	1.009 (0.455–2.234)	0.983
PTPN14	1.116 (0.558–2.235)	0.756	1.439 (0.687–3.016)	0.335
PTPN18	0.875 (0.453–1.693)	0.692	1.001 (0.494–2.031)	0.997
PTPN20	1.697 (0.901–3.194)	0.101	*1.982* (*1.039–3.780*)	*0.038*
PTPN21	0.627 (0.279–1.409)	0.259	0.580 (0.244–1.381)	0.219
PTPN22	0.911 (0.469–1.772)	0.784	0.576 (0.264–1.255)	0.165
PTPN23	0.664 (0.318–1.387)	0.276	0.487 (0.228–1.040)	0.063

Significant P values are expressed in Italics

**Table 4:** Prognostic role of PTPN family genes in STAD

Gene	Univariate analysis		Multivariate analysis	
	HR (95% CI)	P	Adjusted HR (95% CI)	P
PTPN1	1.238 (0.857–1.788)	0.255	1.118 (0.772–1.619)	0.553
PTPN2	1.174 (0.806–1.710)	0.405	1.219 (0.836–1.779)	0.304
PTPN3	0.879 (0.592–1.306)	0.524	0.844 (0.563–1.266)	0.412
PTPN4	0.854 (0.580–1.258)	0.424	0.802 (0.543–1.183)	0.265
PTPN5	*1.680* (*1.162–2.42*9*)*	*0.006*	*1.753* (*1.192–2.577*)	*0.004*
PTPN6	*0.633* (*0.421–0.951*)	*0.028*	*0.606* (*0.402–0.915*)	*0.017*
PTPN7	1.065 (0.731–1.551)	0.743	0.956 (0.650–1.405)	0.819
PTPN9	1.229 (0.853–1.769)	0.269	1.233 (0.855–1.778)	0.263
PTPN11	1.373 (0.951–1.984)	0.091	1.389 (0.957–2.018)	0.084
PTPN12	1.202 (0.811–1.779)	0.359	1.152 (0.778–1.707)	0.479
PTPN13	*1.471* (*1.026–2.109*)	*0.036*	1.431 (0.995–2.058)	0.054
PTPN14	1.341 (0.927–1.937)	0.118	1.350 (0.924–1.973)	0.121
PTPN18	1.158 (0.787–1.705)	0.456	1.213 (0.819–1.796)	0.334
PTPN20	1.185 (0.813–1.727)	0.377	1.389 (0.946–2.040)	0.094
PTPN21	1.237 (0.852–1.797)	0.264	1.258 (0.862–1.836)	0.234
PTPN22	0.913 (0.614–1.358)	0.654	0.844 (0.561–1.271)	0.418
PTPN23	0.954 (0.655–1.390)	0.806	0.998 (0.681–1.463)	0.992

Significant P values are expressed in Italics

**Table 5:** Prognostic role of PTPN family genes in CRC

Gene	Univariate analysis		Multivariate analysis	
	HR (95% CI)	P	Adjusted HR (95% CI)	P
PTPN1	1.492 (0.863–2.579)	0.152	1.476 (0.841–2.588)	0.175
PTPN2	1.136 (0.653–1.975)	0.652	1.009 (0.571–1.783)	0.976
PTPN3	1.022 (0.576–1.814)	0.941	0.912 (0.505–1.649)	0.761
PTPN4	1.003 (0.565–1.780)	0.993	0.903 (0.507–1.609)	0.729
PTPN5	*1.947* (*1.105–3.431*)	*0.021*	1.681 (0.945–2.991)	0.077
PTPN6	0.825 (0.859–2.553)	0.158	1.328 (0.764–2.306)	0.314
PTPN7	1.700 (0.984–2.940)	0.057	*2.043* (*1.164–3.584*)	*0.013*
PTPN9	0.825 (0.428–1.593)	0.567	0.821 (0.424–1.589)	0.558
PTPN11	1.525 (0.698–2.266)	0.446	1.618 (0.885–2.959)	0.118
PTPN12	1.525 (0.885–2.629)	0.129	0.962 (0.539–1.718)	0.896
PTPN13	1.186 (0.661–2.130)	0.568	1.105 (0.607–2.010)	0.744
PTPN14	1.706 (0.978–2.977)	0.06	1.142 (0.646–2.018)	0.648
PTPN18	1.500 (0.852–2.640)	0.16	1.503 (0.835–2.706)	0.174
PTPN20	1.218 (0.699–2.125)	0.487	1.499 (0.853–2.636)	0.16
PTPN21	0.853 (0.443–1.643)	0.634	0.808 (0.418–1.564)	0.528
PTPN22	1.622 (0.945–2.782)	0.079	1.637 (0.949–2.825)	0.076
PTPN23	0.893 (0.483–1.652)	0.719	0.519 (0.268–1.002)	0.051

Significant P values are expressed in Italics

### Function and interaction of PTPN family genes

**Fig. 6:**
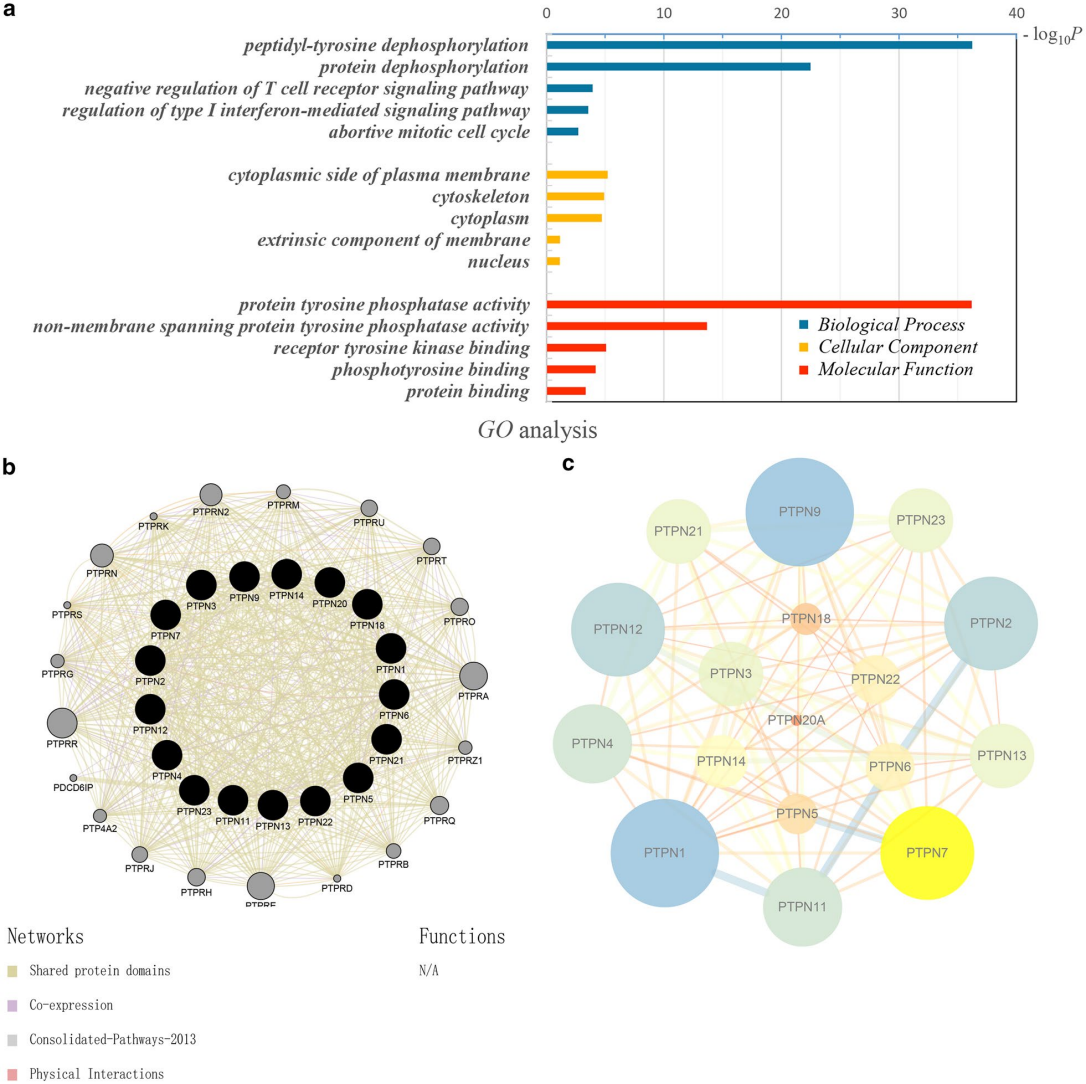
Enrichment and correlation analysis among PTPN family genes. **a** GO analysis of PTPN family genes. The top 5 enriched categories for Biological Process, Cellular Component, and Molecular Function were showed. **b** Gene–gene interaction network among PTPN gene family members. **c** Protein–protein interaction network among PTPN gene family members

GO analysis was basically grouped into three terms including molecular function groups, cellular component groups and biological process groups. The top 5 enriched categories obtained from the analysis results of each group were showed in Fig. 6a. GO analysis revealed that PTPN proteins were mainly related to cytoplasmic side of plasma membrane. PTPN genes exert their functions primarily on peptidyl-tyrosine dephosphorylation and protein tyrosine phosphatase activity. Further, the interaction analysis of PTPN genes at the gene level was performed by GeneMANIA to clarify the correlations among colocalization, shared protein domains, co-expression, prediction and pathways (Fig. 6b). As the protein–protein interaction network of STRING analysis revealed, interrelationships among PTPN gene family members were intricate (Fig. 6c).

### Differential expression of PTPNs in cancer cell lines

We used CCLE to confirm the expression of PTPN1, PTPN2, PTPN4, PTPN12, PTPN21 and PTPN22 in digestive tract cancer cell lines. All of these six PTPNs were differentially expressed in cancer and control groups according to both Oncomine and Ualcan database analysis results. As the Additional file 3 showed, levels of PTPN1, PTPN4 and PTPN12 were higher in esophageal cancer cell lines; PTPN2 and PTPN12 expressions were higher in stomach cancer cell lines; higher PTPN12 and decreased PTPN22 were identified in colorectal cancer cell lines. These results were consistent with our analyses results of Oncomine and Ualcan database. Different from these results, PTPN22 was downregulated in stomach cancer cell lines and PTPN21 were increased in colorectal cancer cell lines, which were inconsistent with the conclusions of Oncomine and Ualcan database. The expression profiles of PTPN21 in colorectal cancer and PTPN22 in stomach cancer warrant further examination.

### Verification of four PTPN expressions in digestive tract cancer tissues

To confirm our analysis results, we then did tissue verification with qRT-PCR experiment. The results revealed that in GC, PTPN12 expression were higher than normal tissues (P = 0.037); in CRC, higher PTPN12 expression and lower PTPN22 expression were observed (P = 0.007 and 0.009, resp.); and marginal difference was found with the expression of PTPN1 in EC (P = 0.074) (Table 6).

**Table 6:** Histological verification of the mRNA expression of PTPN genes

Group	Gene	mRNA expression	
		Mean ± SD	*P* value
EC vs CON	PTPN12	0.026 ± 0.025 vs 0.017 ± 0.016	0.169
	PTPN1	0.017 ± 0.019 vs 0.119 ± 0.218	0.074
GC vs CON	PTPN12	0.010 ± 0.008 vs 0.022 ± 0.022	*0.037*
	PTPN2	0.021 ± 0.011 vs 0.020 ± 0.011	0.646
CRC vs CON	PTPN12	0.001 ± 0.001 vs 0.047 ± 0.084	*0.007*
	PTPN22	0.004 ± 0.006 vs 0.0002 ± 0.0003	*0.009*

Significant P values are expressed in Italics

*CON* adjacent non-tumor tissue

## Discussion

Being significant members of protein tyrosine phosphatases, PTPN regulates tyrosine phosphorylation and dephosphorylation in cellular signal transduction together with protein-tyrosine kinases [4]. There are abundant investigations focusing on the links between individual PTPN family members and diverse neoplasms. However, no report has offered an overview of how PTPN family genes tie up with diverse gastrointestinal tumors up to now. This research for the first time investigated the expression patterns of PTPN genes, and illustrated the association between PTPN family genes and the diagnosis as well as prognosis of digestive tract cancers, which provides deeper insights into the clinical values of all the PTPN genes in various digestive tract cancers.

We used online database Oncomine and Ualcan to explore the PTPN genes expression patterns in digestive tract cancers, ROC models were further established to investigate their diagnostic values. CCLE analysis and human tissue experiment by qRT-PCR were conducted to verify our results. As revealed by our study, expression levels of PTPN5 and PTPN13 were decreased, while PTPN2, PTPN12, and PTPN22 were increased in human STAD. PTPN2 and PTPN12 were further exploited as STAD diagnostic biomarkers. According to previous studies, PTPN2 antagonizes cytokine signaling needed for T cell differentiation, homeostasis, and function by regulating the dephosphorylation and inactivation of Janus-activated kinase (JAK)-1 and JAK-3, and their target substrates signal transducer and activator of transcription (STAT)-1, STAT-3 and STAT-5 in a cell context-dependent manner [23–26]. Recent researches demonstrated that PTPN2 deletion can enhance the effectiveness of anti-tumor immunity [8, 27]. Thus, PTPN2 expression is likely to be exploited as an attractive immunotherapy target in the treatment of cancer, such as STAD. For CRC patients, PTPN3, PTPN7, PTPN11, PTPN12, PTPN13 and PTPN14 were upregulated while PTPN2, PTPN18, PTPN21, and PTPN22 expressions were downregulated according to Oncomine. PTPN12, PTPN21, and PTPN22 were further verified in Ualcan, and proved to be correlated with CRC incidence. PTPN3 was demonstrated to be served as a tumor suppressor gene in CRC [28]. While another study illustrated that elevated PTPN3 expression promotes tumor recurrence and is detrimental to the prognosis of intrahepatic cholangiocarcinoma patients [29]. These findings indicate that regulation mechanisms of PTPN3 in various cancers are complicated. Consistent with our results, Slattery et al. [30] reported that PTPN11 expression is upregulated in CRC. PTPN11 was suggested to impact the tumorigenesis and metastasis of CRC [31] and termed as a potential prognostic marker [32]. The data in one report showed that in patients with gastric B cell non-Hodgkin’s lymphoma, PTPN21 is over expressed [33]. In ESCA, the expression levels of PTPN1, PTPN4, and PTPN12 were increased. Differential expression of PTPN12 in GC and CRC was observed in our histological experiment. ROC results suggested that PTPN12 could serve as a diagnostic biomarker gastrointestinal cancers. In several researches, PTPN12 is characterized as a tumor suppressor which antagonizes EGFR/HER2 signaling [34, 35], which is contrary to our findings. In hepatocellular carcinoma cells, PTPN12 regulates epithelial–mesenchymal transition which contributes to chemoresistance and metastasis [36]. All of these indicate that functions of PTPN12 are different in various cancer types, and comprehensive studies are required to clarify its specific mechanisms.

There may also exist associations between differentially expressed PTPNs and prognosis of digestive tract cancers. To further figure out the associations, we then analyzed the interrelationships between PTPN expressions and the clinical outcomes of digestive tract cancers. As revealed by the current study, PTPN5, PTPN13 and PTPN22 were also associated with clinicopathological parameters of STAD. And decreased expression of PTPN5 and PTPN13 indicated worse OS of STAD patients, while high PTPN6 expression was associated with a favorable STAD OS. It is worth noting that SHP-1 protein encoded by PTPN6 mediates the tumor-suppressive function of TMEFF2 in STAD [37], which indicates expression of PTPN6 might influence the carcinogenesis of STAD patients. PTPN13 has been reported to regulate the resistance of human lung fibroblasts to Fas-induced apoptosis in previous study [38]. Mutated PTPN13 was suggested to be a tumor suppressor gene in colorectal cancer [28]. PTPN22 gene encodes an enzyme called lymphoid-specific tyrosine phosphatase, which functions as a master regulator in the biological process of relevant immune responses [39]. Upregulation of PTPN22 could result in impairment of regulatory T-cell differentiation in patients with non-ST-segment elevation acute coronary syndromes [40]. As for CRC, expression levels of PTPN5, PTPN12, and PTPN14 were correlated with clinicopathological parameters. Besides, elevation of PTPN5 and PTPN7 was significantly associated with increased death hazards of CRC patients respectively. One study presents PTPN12 as a novel candidate that contributes to the heterogeneous susceptibility to colorectal cancer [41]. PTPN5 could regulate tyrosine dephosphorylation needed for the activation of BAK, a noteworthy cell-death mediator in apoptosis [42]. It was once reported that PTPN14 regulated phosphorylation of p130Cas Y128 plays a crucial role in colorectal carcinogenesis [43]. And mutated PTPN14 is suggested to be a tumor suppressor gene for colorectal cancer, regulating cellular pathways that are appropriate for therapeutic intervention [28]. A recent study proved that PTPN14 suffices to inhibit migration and invasion of metastatic cancer cells [44]. In a xenograft breast cancer model, PTPN14 acts as a suppressor of metastasis of triple-negative breast cancer cells [45]. In our analysis, PTPN5 and PTPN14 expression was associated with the clinicopathological parameters of both STAD and CRC. However, differential expressions of PTPN5 in GC and controls, and PTPN14 in CRC and controls were observed only in Oncomine database, but were not further verified in Ualcan database. Taken together, we hypothesized that PTPN5 and PTPN14 mainly function in the process of tumor progression, and its correlation with the risk of cancer requires further evaluation and experiments. As for ESCA, our analysis indicated that only PTPN20 expression was related with a worse OS of patients. However, former research indicated that PTPN12 may serve as a potential prognostic indicator for esophagus cancer patients [46]. In our study, some PTPNs were associated with overall survival of cancer patients, but not associated with clinicopathological parameters. As we all know, variables including patient-level, tumor-level and environment-level factors all exert great influence on survival outcomes of cancer patients, which have been illustrated by multiple laboratory evidence and clinical experience. Thus it can be ratiocinated that intrinsic interactions may exist between PTPNs and other clinical factors and consequently affect the prognosis of cancer. In order to understand the functions of PTPNs in gastrointestinal cancers better, investigations concerning more clinicopathological parameters are requisite in the future.

In this study, GO analysis revealed that PTPN proteins were mainly related to cytoplasmic side of plasma membrane. PTPN genes exert their functions primarily on peptidyl-tyrosine dephosphorylation and protein tyrosine phosphatase activity, which was illustrated by dozens of publications. Substrate-trapping and biochemical analyses demonstrated that PTPN22 mainly dephosphorylates the tyrosine residues of SFKs and SFK substrates including the E3ubiquitin ligase c-Cbl57-59 and the T cell signaling protein Zap70 [47–49]. PTPN5 was reported to mediate internalization and phosphorylation of AMPA receptors after metabotropic glutamate receptor stimulation [50]. In addition, the results of interaction network analysis at gene and protein levels further indicated that PTPN members and other genes comprehensively interact with each other. One experiment suggested that both PTPN1 and PTPN2 target protein mediator of IRF3 activation for dephosphorylation at Y245 [51]. PTPN2 and PTPN22 were demonstrated negatively regulating T cell receptor signaling by dephosphorylating lymphocyte-specific protein tyrosine kinase [47, 52]. Multiple efforts demonstrated that through binding and inactivating the mitogen activated protein kinase Erk2 and p38, PTPN5 and PTPN7 could negatively regulate cell proliferation as well as differentiation [53–56]. Promoter hypermethylation of PTPN6 and PTPN13 was reported to inhibit the progression of diffuse large B cell lymphomas [57]. In our study, PTPN5 and PTPN7 were found to be correlated with prognosis of colon adenocarcinoma patients, while PTPN6 and PTPN13 were statistically associated with the prognosis of STAD. All of these implied that PTPN members could function through alliance mechanisms in many diseases, including gastrointestinal cancers.

## Conclusions

In summary, findings of our study illustrated the expression status as well as diagnostic and prognostic values of PTPN members in digestive tract cancers. The results indicated that several PTPN members were differentially expressed and related to clinical outcomes of patients with digestive tract cancers. Especially, upregulation of PTPN12 was correlated with the incidence of ESCA, STAD and CRC. Differential expression of PTPN12 in GC and CRC, and PTPN22 in CRC were presented in our histological verification experiment. Future well-designed investigations are required to elucidate the significance of our findings and thus develop the clinical utility of PTPNs.

## Supplementary information

**Additional file 1: Table S1.** The list of primers; **Table S2.** Association of PTPN family genes expression with clinicopathological parameters of ESCA; **Table S3.** Association of PTPN family genes expression with clinicopathological parameters of STAD; **Table S4.** Association of PTPN family genes expression with clinicopathological parameters of CRC.

**Additional file 2.** Diagnostic value of differentially expressed PTPNs in digestive tract cancers. **a** ROCs of PTPN1/4/12 levels in ESCA, showing that elevated PTPN1/12 levels were correlated with ESCA incidence. **b** ROCs of PTPN2/12/22 levels in STAD, showing that elevated PTPN2/12 levels were correlated with STAD incidence. **c** ROCs of PTPN12/21/22 levels in CRC, showing that elevated PTPN12 levels and decreased PTPN21/22 levels were correlated with CRC incidence.

**Additional file 3.** Expression profiles of certain PTPNs in cancer cell lines. **(a)** PTPN1 **(b)** PTPN2 **(c)** PTPN4 **(d)** PTPN12 **(e)** PTPN21 **(f)** PTPN22.

## Data Availability

The datasets analyzed during the current study are available in the TCGA repository (https://cancergenome.nih.gov/). The authors declare that the data supporting the findings of this study are available within the article.

## Abbreviations

PTPs: Protein tyrosine phosphatases
PTPN: Non-receptor protein tyrosine phosphatase
EC: Esophagus cancer
GC: Gastric cancer
CRC: Colorectal cancer
TCGA: The Cancer Genome Atlas
ESCA: Esophageal carcinoma
STAD: Stomach adenocarcinoma
COAD: Colon adenocarcinoma
READ: Rectum adenocarcinoma
ROC: Receiver operating characteristics
GO: Gene Ontology
DAVID: Database for Annotation, Visualization and Integrated Discovery
GeneMANIA: Gene Multiple Association Network Integration Algorithm
STRING: Search Tool for the Retrieval of Interacting Genes Database
PPI: Protein–protein interaction
CCLE: Broad Institute Cancer Cell Line Encyclopedia
OS: Overall survival
JAK: Janus-activated kinase
STAT: Signal transducer and activator of transcription
